# Clinical application of unilateral intramedullary K-wire occupying technique for flexible reduction in pediatric supracondylar humeral fractures

**DOI:** 10.3389/fsurg.2026.1879655

**Published:** 2026-08-26

**Authors:** Wenqiang Xu, Wei Wang, Xiulin Ma, Yongfei Fan, Jianqiang Zhang, Chaoyu Liu, Haiyang Yu

**Affiliations:** Department of Orthopaedics, Affiliated Fuyang People's Hospital of Anhui Medical University, Fuyang, Anhui, China

**Keywords:** pediatric supracondylar humeral fracture, K-wire, intramedullary occupying technique, ultrasound-guided reduction, retrospective study

## Abstract

**Objective:**

To investigate the clinical efficacy of unilateral Kirschner wire (K-wire) intramedullary occupying technique for flexible reduction combined with ultrasound guidance in the treatment of pediatric supracondylar humeral fractures.

**Methods:**

A retrospective analysis was conducted on 41 patients with Gartland type III and IV pediatric supracondylar humeral fractures treated in our hospital from January 2024 to April 2025. Among them, 21 patients underwent closed reduction assisted by the unilateral K-wire intramedullary occupying technique (observation group), and 20 patients received traditional closed reduction (control group). No statistically significant differences were observed between the two groups in age, sex, injury cause, fracture side, Gartland classification, or time from injury to surgery (*P* > 0.05). Operative time, intraoperative fluoroscopy frequency, fracture healing time, complication rate, and elbow function assessed by Flynn score, Baumann angle, and carrying angle at the last follow-up were compared between the two groups.

**Results:**

All fractures in both groups underwent ultrasound-guided closed reduction. The observation group had significantly shorter operative time and fewer intraoperative fluoroscopic acquisitions than the control group (*P* < 0.05). All patients were followed up for 12–24 months (data cutoff: May 31, 2026), and imaging showed fracture healing in both groups, with no significant difference in healing time (*P* = 0.920). At the last follow-up, the Flynn score indicated an excellent/good rate of 100% in both groups. No significant differences were found between the healthy and affected sides in Baumann angle or carrying angle in either group (*P* > 0.05). Pin site irritation occurred in 4 cases in the observation group and 3 cases in the control group, with no significant difference (*P* = 1.000).

**Conclusion:**

For pediatric Gartland type III and IV supracondylar humeral fractures with significant lateral displacement, both ultrasound-guided closed reduction techniques achieve satisfactory clinical outcomes. However, the unilateral K-wire intramedullary occupying technique is relatively simpler, with shorter operative time and fewer fluoroscopic acquisitions, making it a viable surgical option for such fractures.

## 1 Introduction

Supracondylar humeral fractures are among the most common upper extremity fractures in children (1). Closed reduction and percutaneous Kirschner wire (K-wire) fixation have become the standard surgical treatment for such fractures (2, 3). For pediatric orthopedic surgeons, conventional closed reduction and percutaneous K-wire fixation can manage most displaced supracondylar humeral fractures. However, in some children, closed reduction is difficult, particularly in cases of radial deviation or multidirectional instability. Conventional closed reduction methods may fail to achieve satisfactory results, leading to compromised outcomes or conversion to open reduction (4).

Based on intraoperative observations, for fractures that remain difficult to reduce after initial manipulation, we propose inserting one K-wire percutaneously from the radial or ulnar side, advancing it at a certain oblique angle through the fracture site into the proximal medullary canal. By utilizing the elastic intramedullary occupying effect of the K-wire, unilateral column stability is restored, and one side of the fracture is reduced. The other side remains elastically stabilized, allowing targeted manual reduction according to the direction of displacement. After satisfactory reduction is achieved, percutaneous K-wire fixation is performed.

This study retrospectively analyzed 41 patients with Gartland type III and IV pediatric supracondylar humeral fractures treated in our hospital from January 2024 to April 2025. Compared with traditional closed reduction and percutaneous K-wire fixation, we evaluated the advantages of using the unilateral K-wire intramedullary occupying effect to assist reduction, providing a reference for surgical decision-making. The report is as follows.

## 2 Clinical data

### 2.1 Inclusion and exclusion criteria

Inclusion criteria: (1) age ≤ 14 years; (2) preoperative or intraoperative imaging diagnosis of Gartland type III or IV supracondylar humeral fracture; (3) follow-up duration ≥ 12 months.

Exclusion criteria: (1) open fracture; (2) pathological fracture; (3) old fracture.

### 2.2 General data

From January 2024 to April 2025, 41 children meeting the inclusion criteria were enrolled. Among them, 21 patients underwent closed reduction assisted by the unilateral K-wire intramedullary occupying effect (observation group), and 20 patients received traditional closed reduction (control group). No significant differences were observed between the two groups in age, sex, fracture side, Gartland classification, or time from injury to surgery (*P* > 0.05). All injuries were caused by falls. The groups were comparable. See Table 1.

**Table 1:** Comparison of general data between the two groups.

Group	Gender (*n*)		Age (years, mean ± SD)	Side (*n*)		Time from injury to surgery (days, mean ± SD)	Fracture classification (Gartland) (*n*)		Milking maneuver performed (*n*)
	Boy	Girl		Left	Right		Ⅲ	Ⅳ	
Observation（*n* = 21）	15	6	7.14 ± 1.93	12	9	2.14 ± 0.85	15	6	1
Control （*n* = 20）	14	6	7.30 ± 1.75	11	9	2.10 ± 0.85	15	5	1
Statistic (F/χ²)	0.010		0.074	0.019		0.026	0.08		-^a^
*P* value	0.920		0.787	0.890		0.873	0.929		1.000

a Fisher’s exact test was used for this column, no χ² value generated.

This study was approved by the Ethics Committee of Fuyang People's Hospital Affiliated to Anhui Medical University [Ethics Approval No.: 2022(19)]. All patients and their legal guardians were informed preoperatively and signed informed consent forms.

### 2.3 Surgical methods

All major surgical steps were performed by the same surgeon. The patient was placed in a supine position under general anesthesia. Lead aprons were used to protect vital glands. Sterile draping was applied. The C-arm receiver was placed close to the operating table edge for easy access. An ultrasound device was connected, and the probe cable was wrapped with a sterile plastic cover. The surgeon and assistant performed adequate traction on the fracture ends to separate them. If there were signs of skin dimpling, ecchymosis, or bone fragment interlocking, the “milking” maneuver (5) was attempted to restore the length of the fracture.

Intraoperative ultrasound was employed as the primary imaging modality to guide reduction for the following reasons: (1) it provides real-time, multi-planar visualization without ionizing radiation, thereby minimizing radiation exposure to pediatric patients and surgical staff; (2) it has been shown to be as reliable as fluoroscopy for evaluating fracture reduction, with comparable measurement accuracy; (3) it offers superior soft tissue visualization, allowing real-time identification and protection of the ulnar nerve during medial pin placement; (4) it enables continuous dynamic monitoring throughout the reduction process, avoiding repeated fluoroscopic acquisitions and thus shortening operative time; and (5) it effectively assesses sagittal plane reduction quality by visualizing the anterior humeral line–capitellum relationship. Fluoroscopy was reserved for final confirmation of reduction and fixation.

#### 2.3.1 Observation group

**Figure 1:**
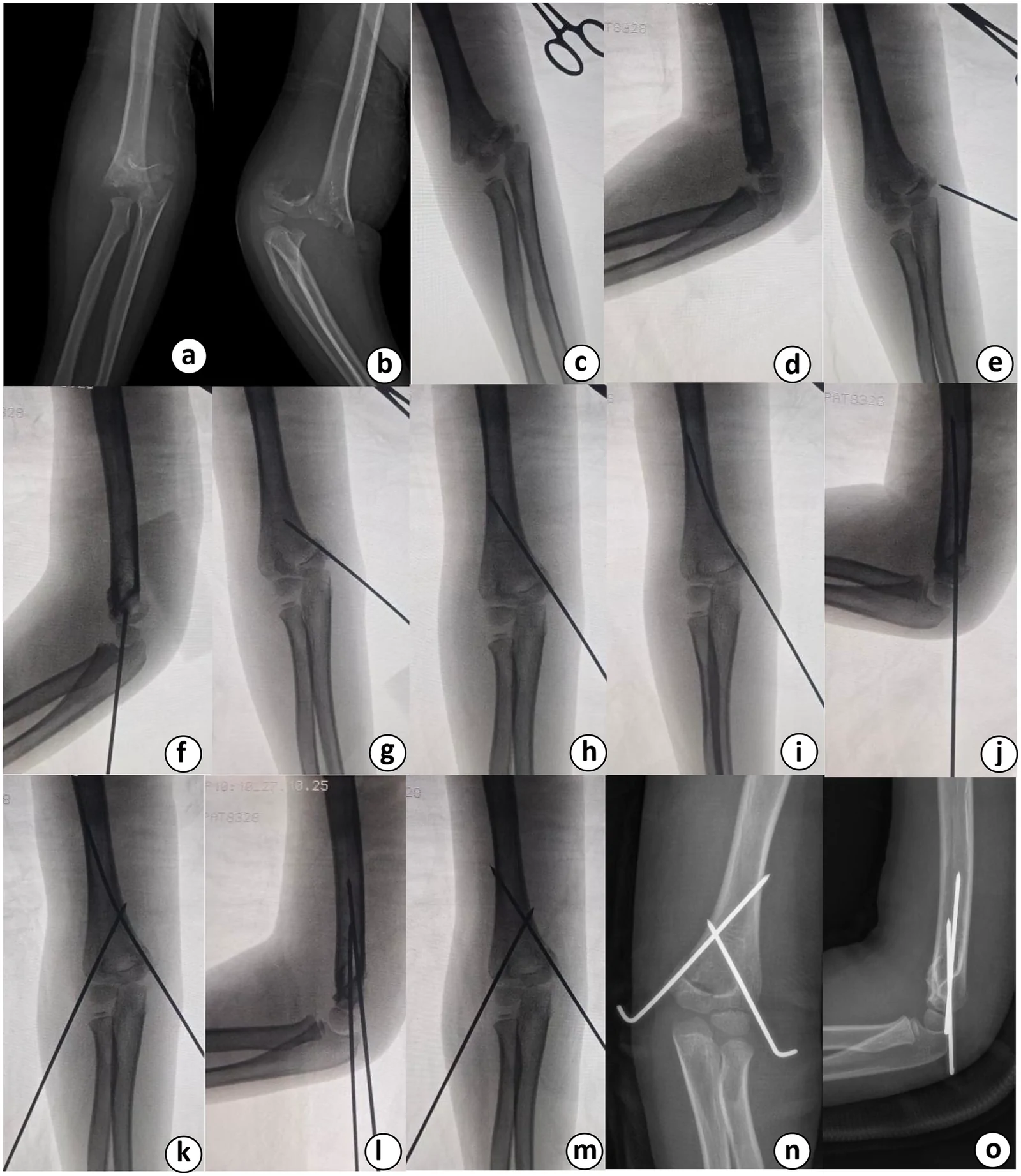
A 6-year-old boy with a Gartland type III supracondylar humeral fracture (ulnar deviation pattern). **(a,b)** Preoperative anteroposterior (AP) and lateral radiographs showing the fracture. **(c,d)** Intraoperative fluoroscopy after initial closed manipulation: AP and lateral views showing persistent ulnar deviation. **(e,f)** Fluoroscopic localization of the ulnar entry point. **(g–i)** Gradual advancement of the ulnar K-wire into the medullary canal, achieving intramedullary occupying and correcting ulnar deviation. **(j)** Lateral fluoroscopic view: further reduction performed if needed. **(k,l)** Satisfactory reduction achieved, followed by radial-side K-wire fixation. **(m)** Retraction of the ulnar K-wire and reinsertion at a less steep angle to engage the opposite cortex, enhancing stability. **(n,o)** Postoperative AP and lateral radiographs of the elbow. This figure demonstrates the complete standardized surgical workflow of the technique.

**Figure 2:**
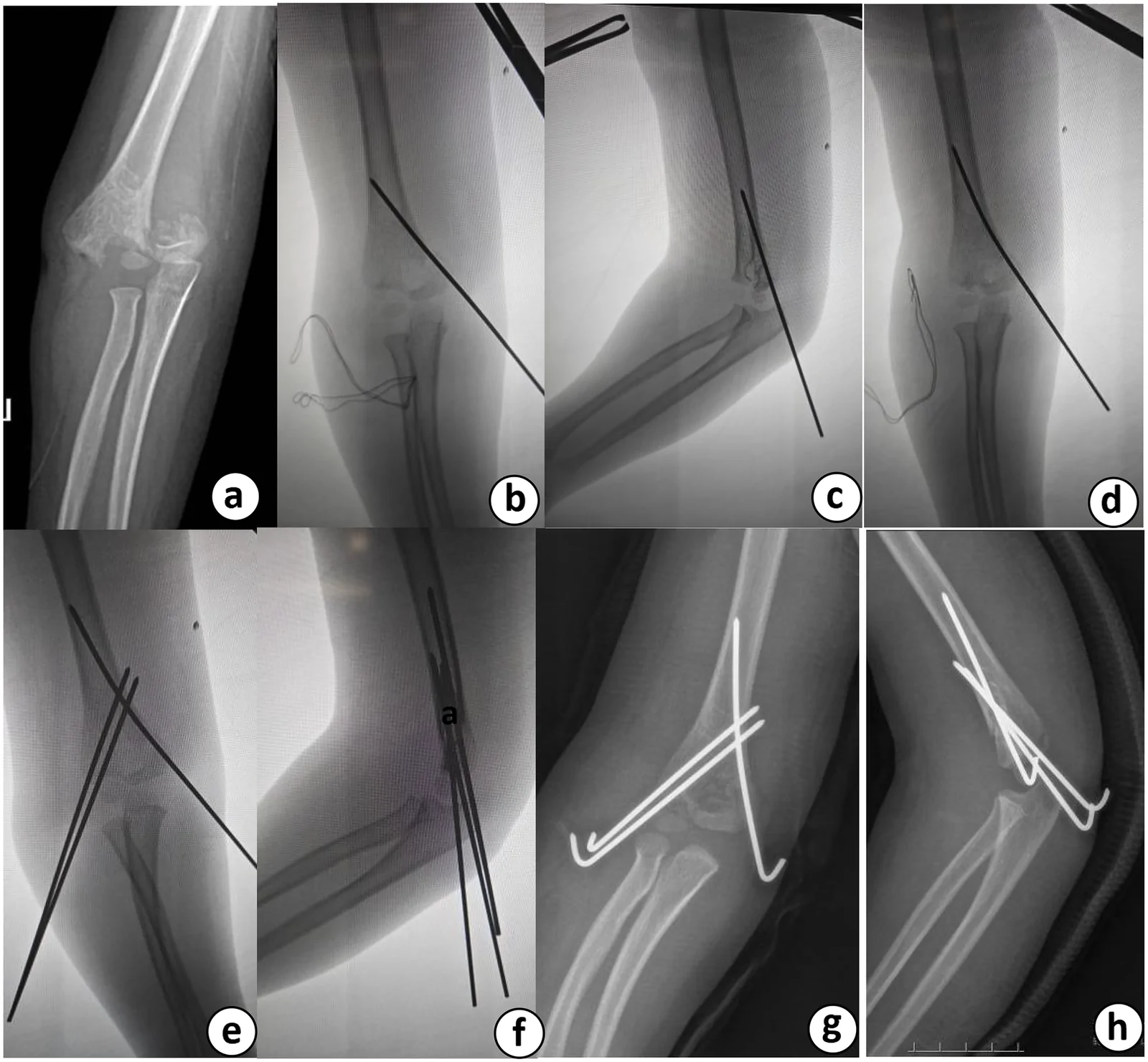
A 5-year-old boy with a Gartland type III supracondylar humeral fracture with an oblique fracture line running from superomedial to inferolateral. **(a)** Preoperative radiograph showing the fracture. **(b,c)** After initial closed manipulation, fluoroscopic localization of the ulnar entry point and K-wire insertion. **(d)** Gradual advancement of the ulnar K-wire into the medullary canal, correcting ulnar deviation. **(e,f)** Satisfactory reduction achieved, followed by radial-side K-wire fixation. **(g,h)** Postoperative AP and lateral radiographs of the elbow. This figure highlights the management of oblique fracture patterns, where the K-wire is inserted on the higher side of the fracture line to optimize compressive stress and shear force direction.

The ultrasound probe was used to examine the fracture ends from four directions: medial ulnohumeral joint, lateral radiohumeral joint, anterior radioulnar joint, and anterior ulnohumeral joint (6) to clarify the direction of displacement. For radial deviation, the elbow was flexed and externally rotated. A 2.0 mm K-wire was drilled from the lateral condyle of the distal humerus under fluoroscopic guidance to ensure the wire was parallel to the axis of the distal fragment and as central as possible. The insertion angle was minimized. Under extension view, the wire was advanced through the fracture site into the proximal medullary canal. The elastic force of the K-wire corrected the radial deviation. Ultrasound was repeated to assess residual displacement. If ulnar rotation remained, targeted manual reduction was performed. After confirmation of reduction, additional K-wires were inserted percutaneously in a crossed configuration. For ulnar-sided insertion, the ultrasound probe was placed over the medial aspect of the ulnohumeral joint to identify the medial epicondyle and ulnar nerve groove. The intramedullary K-wire was either retained or partially withdrawn and reinserted at a less steep (more horizontal) angle to engage the opposite cortex, thereby enhancing stability. For ulnar deviation, the same procedure was performed starting from the ulnar side. Typical cases are shown in Figures 1, 2.

#### 2.3.2 Control group

Ultrasound was used to identify the direction of displacement. Reduction was achieved by continuous longitudinal traction followed by gradual elbow flexion to 90°, correcting posterior and lateral displacement. The entire reduction process was guided by ultrasound. After successful reduction, the elbow was flexed, and the olecranon was pushed. Two K-wires were inserted from the radial side. Before ulnar insertion, the ulnar nerve was identified using ultrasound. One K-wire was then inserted from the medial epicondyle obliquely upward through the fracture line. After crossed fixation, additional K-wires were used as needed. Final reduction and fixation were confirmed by fluoroscopy.

After completion of the procedure, all K-wires were bent and cut short. The wire ends were wrapped with gauze, and a sterile dressing was applied. A long-arm cast was applied with the elbow flexed at 90° and the forearm in neutral rotation.

### 2.4 Postoperative management and outcome evaluation indicators

Postoperative management was identical for both groups: limb sensation, movement, and swelling were monitored. Regular outpatient follow-up was scheduled. At 4–6 weeks postoperatively, radiographs were taken. If continuous callus formation was observed, the cast was removed, and K-wires were extracted followed by elbow function exercises. Operative time (defined from the start of fluoroscopic/ultrasound evaluation to completion of bicortical fixation), fluoroscopy frequency, fracture healing time, elbow function according to the Flynn score, Baumann angle, carrying angle, and complications during follow-up were recorded. The follow-up data collection cutoff date was May 31, 2026, ensuring that all enrolled patients had completed a minimum of 12 months of follow-up.

### 2.5 Statistical analysis

SPSS 26.0 was used for statistical analysis. Categorical data were expressed as *n* (%) and analyzed using the χ² test or Fisher's exact test. Continuous data were expressed as mean ± SD and analyzed using one-way ANOVA. *P* < 0.05 was considered statistically significant.

## 3 Results

Closed reduction was successful in all patients. The observation group had significantly shorter operative time and fewer intraoperative fluoroscopic acquisitions than the control group (*P* < 0.05). All patients were followed up for 12–24 months (data cutoff: May 31, 2026). All fractures healed without significant difference in healing time (*P* > 0.05). At the last follow-up, the excellent/good rate according to the Flynn score was 100% in both groups. Pin site irritation occurred in 4 cases in the observation group and 3 cases in the control group, with no significant difference. No significant differences were found in Baumann angle or carrying angle between the healthy and affected sides in either group. No cases of reduction loss, nerve injury, elbow stiffness, or cubitus varus were observed during follow-up. See Tables 2, 3.

**Table 2:** Comparison of perioperative indicators between the two groups.

group	operation time minutes, mean ± SD	Fluoroscopy frequency (mean ± SD)	Fracture healing time week, mean ± SD	Incidence of complications
observation group	24.76 ± 3.70	8.95 ± 2.11	4.43 ± 0.68	4/21
control group	45.50 ± 7.59	12.85 ± 3.33	4.45 ± 0.69	3/20
F/χ² Value	125.523	20.264	0.010	-^a^
*P* Value	＜0.01	＜0.01	0.920	1.000

a Fisher’s exact test was used for this column, no χ² value generated.

**Table 3:** Imaging indicators of patients in both groups at the last follow-up.

Group	Indicator	Healthy side (°, mean ± SD)	Affected side (°, mean ± SD)	F value	*P* value
Observation	Baumann angle	71.52 ± 4.15	71.71 ± 4.33	0.358	0.722
	Carrying angle	9.48 ± 2.71	9.76 ± 2.51	0.503	0.618
Control	Baumann angle	72.35 ± 4.09	72.45 ± 4.12	0.367	0.715
	Carrying angle	9.95 ± 2.93	10.15 ± 2.70	0.399	0.692

## 4 Discussion

Pediatric supracondylar humeral fractures vary in displacement patterns depending on anatomy, injury mechanism, and force direction, leading to different fracture types (7–9). For those requiring surgery, achieving closed anatomical reduction, stable fixation, and avoiding complications are the ideal goals (10, 11). Extension-ulnar deviation fractures are the most common and can often be managed with conventional closed reduction and percutaneous pinning. However, fractures with radial deviation, flexion, multidirectional instability, or iatrogenic disruption of the periosteal hinge during reduction attempts are more challenging. Recently, new surgical techniques have been reported to increase the success rate of minimally invasive treatment for such fractures (12–14).

We found that the unilateral K-wire intramedullary occupying technique achieved excellent reduction results. By inserting a K-wire into the proximal medullary canal, the elastic force of the wire provides stress at the fracture site, correcting radial/ulnar deviation. This provides preliminary stability, preventing complete redisplacement when traction is released or the limb position changes. Since the K-wire does not engage the opposite cortex, only relative stability is achieved, allowing further manual adjustment on the contralateral side due to residual mobility in the coronal and sagittal planes. Intraoperative ultrasound guidance allows the surgeon and assistant to hold the fracture ends and monitor the reduction in real time, avoiding repeated fluoroscopy and wire insertion, thereby shortening operative time.

The mechanical principle of this technique can be summarized as “elastic correction dominant, physical occupying supportive”: the 2.0 mm K-wire, inserted obliquely across the fracture into the proximal medullary canal, undergoes elastic deformation and generates sustained recoil force that actively corrects radial or ulnar deviation; simultaneously, the occupying effect limits coronal translation of the fragments, providing temporary stability and preventing reduction loss. This “flexible control” allows the surgeon to perform targeted manual adjustments on residual displacement while avoiding the difficulty of correction associated with fully rigid fixation. For radial deviation fractures, the procedure is performed symmetrically—the entry point is simply switched from the ulnar side to the radial side, and the intramedullary occupying effect corrects the radial deviation in the reverse direction. The technical workflow is identical to that illustrated in Figure 1.

For radial deviation fractures, rotational displacement and fracture “straddling” often occur, making reduction difficult. Sustained longitudinal traction and gentle lateral condylar pushing are required to disengage the fragments. With continued traction and proximal stabilization, the olecranon is pushed and the elbow is gradually flexed. Once ultrasound confirms initial contact and reduction, a radial-side K-wire is inserted into the proximal medullary canal to correct radial deviation via the elastic occupying effect. The intramedullary wire provides temporary fixation, allowing gentle ulnar-side manipulation to achieve further reduction in the coronal and sagittal planes.

For oblique fractures, the K-wire is inserted on the higher side of the fracture line, using the wire's rebound effect to compress the fragments, generating compressive stress and directing shear forces distally. This improves stability, preventing significant displacement during manipulation. Traction and pushing forces allow the fragments to slide slowly along the fracture line. After satisfactory reduction, K-wires are inserted on the lower side of the fracture line. The occupying K-wire is then retracted and reinserted as perpendicularly as possible to the fracture line to enhance stability.

For multidirectionally unstable fractures with complete disruption of the periosteal hinge, conventional reduction and maintenance are often ineffective. Repeated manipulation worsens instability, and repeated pinning decreases hold, leading to compromise or conversion to open reduction (15). Open reduction is associated with difficulty locating reduction landmarks due to prior manipulation, increased trauma, longer operative time, and higher complication rates (16). Various strategies have been proposed to address this dilemma. Leitch and Novais et al. (8, 17) used a pre-inserted K-wire as a joystick and moved the C-arm rather than rotating the patient's arm. Pei et al. (13) used fluoroscopic localization and K-wire leverage to assist reduction. Wei et al. (14) described an olecranon joystick technique by inserting one K-wire connecting the olecranon, elbow joint, and distal fragment, followed by manipulation of the medial/lateral condyles, and elevating/depressing or rotating the forearm to reduce unstable fragments. In our technique, K-wire insertion temporarily restores unilateral periosteal stability, connecting the proximal and distal fragments as a single unit. Using this stability, the contralateral fragment is then reduced. For flexion-type fractures, after distal pin insertion, the elbow is slightly extended to allow the wire to enter the proximal medullary canal. Ultrasound provides real-time assessment of reduction, avoiding redisplacement due to changes in limb position, especially rotation, without moving the C-arm. Compared with the control group, the observation group had significantly fewer fluoroscopic acquisitions and shorter operative time, consistent with the findings in the Results section.

**Precautions for this technique:**

1. Wire elasticity increases with diameter. 1.5 mm K-wires are too flexible and provide insufficient elastic force for reduction. A 2.0 mm wire is recommended; for larger children, a 2.5 mm wire may be used.
2. Full fracture length must be restored before pin insertion to avoid interlocking. The “milking” maneuver (5) can be used to disengage fragments.
3. For ulnar deviation fractures requiring ulnar pin insertion, ultrasound can identify the ulnar nerve (18), or the nerve can be palpated and protected with the thumb (19). The entry point should be as superior and anterior as possible on the medial epicondyle to avoid iatrogenic injury.
4. The K-wire must be aligned with the axis of the distal fragment and ideally centered within the medullary canal to maximize lateral stress and self-reduction in the sagittal plane.
5. During follow-up, some intramedullary K-wires (lacking bicortical anchorage) showed loosening or back-out. Therefore, after reduction, the wire can be partially withdrawn and reinserted at a less steep (more horizontal) angle to engage the opposite cortex, improving stability and reducing irritation. If stability is already confirmed, this adjustment is not mandatory; good postoperative bandaging can minimize loosening.

In summary, compared with traditional closed reduction, the unilateral K-wire intramedullary occupying technique for flexible reduction, combined with intraoperative ultrasound guidance, reduces operative time and fluoroscopy frequency, making it a viable option for closed reduction of pediatric supracondylar humeral fractures.

## 5 Limitations

This study has several limitations. First, it is a retrospective analysis, which may be affected by data completeness and potential bias. Second, the sample size is relatively small, and the follow-up duration is limited, which may affect statistical power and long-term outcome assessment. Third, this technique was not directly compared with other existing techniques (e.g., joystick technique), so differences in healing time or postoperative displacement rate remain unclear. Fourth, the illustrative cases in this study primarily demonstrate the ulnar deviation pattern, as the available radial deviation cases did not meet the image quality standards for clear procedural demonstration; however, the technical principles for radial deviation are symmetrical and have been adequately described in the text. Future studies with larger sample sizes, longer follow-up, and appropriate control groups are needed to further validate and optimize the clinical application of this technique.

## Data Availability

The original contributions presented in the study are included in the article/Supplementary Material, further inquiries can be directed to the corresponding authors.
